# PROMOTING EQUITY IN DEMENTIA BEHAVIORAL CLINICAL TRIALS: INSIGHTS FROM THE SOCIO-ECOLOGICAL MODEL

**DOI:** 10.1093/geroni/igae098.0872

**Published:** 2024-12-31

**Authors:** Ryan Mace, Joshua Cohen, Christine Ritchie, Stephen Bartels, Olivia Okereke, Bettina Hoeppner, Judson Brewer, Ana-Maria Vranceanu

**Affiliations:** Massachusetts General Hospital/Harvard Medical School, Boston, Massachusetts, United States; Massachusetts General Hospital, Boston, Massachusetts, United States; Mass General Hospital and Harvard Medical School, Boston, Massachusetts, United States; Massachusetts General Hospital/Harvard Medical School, Boston, Massachusetts, United States; Massachusetts General Hospital, Boston, Massachusetts, United States; Massachusetts General Hospital/Harvard Medical School, Boston, Massachusetts, United States; Brown University Medical School/Brown University School of Public Health, Providence, Rhode Island, United States; Massachusetts General Hospital, Boston, Massachusetts, United States

## Abstract

Alzheimer’s Disease and Related Dementias (ADRD) is exacerbated by social determinants of health, which limit equitable access to effective behavioral interventions. ADRD disparities are widened by clinical trials that fail to recognize and address socio-ecological barriers to health behavior change, thereby reducing their efficacy and reach within diverse older populations. To address this inequity, our qualitative study aimed to: (1) identify socio-ecological barriers to ADRD prevention from healthcare professionals’ viewpoints, and (2) recommend strategies to mitigate these barriers within the context of a ADRD prevention program (My Healthy Brain) and its trial protocol. We conducted 5 focus groups with multidisciplinary healthcare professionals involved in geriatric care (N=26, M experience > 17 years) from diverse clinics within an academic medical center and affiliated community health clinics. Using the Socio-Ecological Model (SEM), we identified individual, interpersonal, institutional, community, and societal barriers. The Expert Recommendations for Implementing Change (ERIC) framework guided the proposal of evidence-based strategies to overcome these barriers. Professionals identified multi-level barriers, including limited resources, linguistic and technological challenges, provider dismissiveness, conflicting institutional priorities, minority underrepresentation, and societal biases towards biomedical treatments. Recommended strategies included modifications to My Healthy Brain to improve accessibility, enhanced provider training and support, integration of interventions within clinical operations, building community partnerships, and rectifying societal misconceptions and biases. Our findings advocate for a socio-ecological approach in ADRD prevention, emphasizing the importance of recognizing and addressing the contextual factors influencing behavior change. The application of SEM and ERIC frameworks serves to bolster equity in ADRD clinical trials.

